# Development of an Improved Peroxidase-Based High-Throughput Screening for the Optimization of D-Glycerate Dehydratase Activity

**DOI:** 10.3390/ijms21010335

**Published:** 2020-01-03

**Authors:** Benjamin Begander, Anna Huber, Manuel Döring, Josef Sperl, Volker Sieber

**Affiliations:** 1 Chair of Chemistry of Biogenic Resources, Campus Straubing for Biotechnology and Sustainability, Technical University of Munich, Schulgasse 16, D-94315 Straubing, Germany; 2 Catalysis Research Center, Technical University of Munich, 85748 Garching, Germany; 3 School of Chemistry and Molecular Biosciences, The University of Queensland, St. Lucia, QLD 4072, Australia

**Keywords:** enzyme engineering, high-throughput screening, biocatalysis, dehydratases

## Abstract

Successful directed evolution examples span a broad range of improved enzyme properties. Nevertheless, the most challenging step for each single directed evolution approach is an efficient identification of improved variants from a large genetic library. Thus, the development and choice of a proper high-throughput screening is a central key for the optimization of enzymes. The detection of low enzymatic activities is especially complicated when they lead to products that are present in the metabolism of the utilized genetic host. Coupled enzymatic assays based on colorimetric products have enabled the optimization of many of such enzymes, but are susceptible to problems when applied on cell extract samples. The purpose of this study was the development of a high-throughput screening for D-glycerate dehydratase activity in cell lysates. With the aid of an automated liquid handling system, we developed a high-throughput assay that relied on a pre-treatment step of cell extract prior to performing the enzymatic and assay reactions. We could successfully apply our method, which should also be transferable to other cell extract-based peroxidase assays, to identify an improved enzyme for the dehydration of D-glycerate.

## 1. Introduction

The increasing scarcity of fossil resources as well as an urgent demand for a reduction of global warming has enhanced the interest in sustainable products. Consequently, novel production processes are needed, that allow the conversion of biomass to bulk and fine chemicals by simultaneously working under the conditions of green chemistry [1]. To reach such processes, catalysis, and especially biocatalysis, is a central key. Enzymes, the drivers of biocatalysis, work under energy-efficient and sustainable reaction conditions and are increasingly applied in industrial processes as they allow a combination of high turnover numbers with high selectivity [2]. Despite these advantages, many enzymes isolated from natural origins show limited technical applicability due to low activity, low stability, or other parameters that do not allow economically feasible industrial processes [3]. In such cases, researchers usually apply enzyme-engineering techniques to overcome those limitations or even develop enzymes for chemical reactions that are naturally not catalyzed [4]. Rational design and directed evolution are the adopted strategies for such endeavors. While rational design intrinsically depends on the presence of a crystal structure and an understanding of the catalytic mechanism of an enzyme, directed evolution provides a powerful approach to optimize enzymes without a clearer picture of the molecular requirements of enzyme catalysis [5]. Successful examples from the last two decades span a broad range of improved properties like activity, thermostability, substrate specificity, product specificity, and enantioselectivity [6,7,8,9,10]. Nevertheless, the most challenging step for each single directed evolution approach is an efficient identification of improved variants from a large genetic library. Thus, the development and choice of a proper high-throughput screening is a central requirement for the optimization of enzymes [11,12]. Successful methods generally show sufficient sensitivity, high repeatability, and low variation among each sample, as well as a high discrimination between positive and negative controls and the target signal. Above that, the designed method must allow an economically feasible screening of a large number of samples [3]. Thus, current advances in enzyme engineering rely heavily on the particular analytical screening method used and on their integration, e.g., into microplate assays that, in combination with automated liquid handling, enable a fast processing of a large number of enzyme variants.

**Scheme 1:**
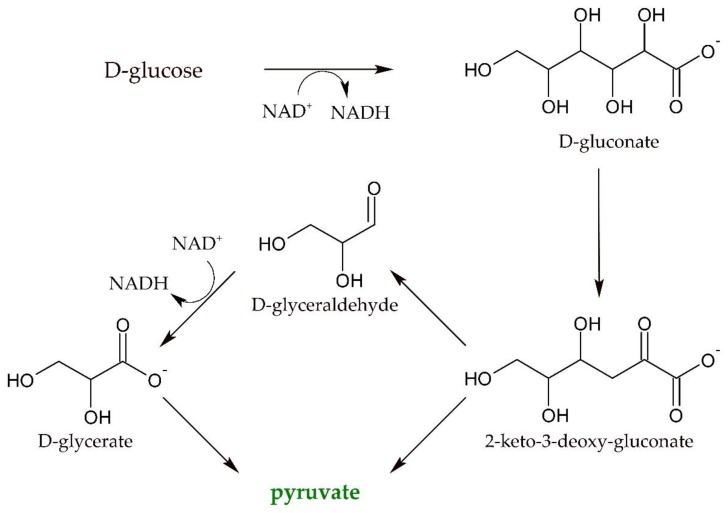
Reaction scheme for the synthesis of the central intermediate pyruvate starting from D-glucose [15].

Besides optimizing single enzymes for one-step biotransformations, multi-step cascades have emerged as a powerful technique, albeit often posing the need for simultaneous optimization of several enzymes [13,14]. Despite that, enzymatic cascades bring several advantages like easy downstream processing and superior reaction control compared to whole-cell approaches. In order to excel whole-cell processes that intrinsically comprise, e.g., balanced cofactor recycling systems, increasingly complex enzyme cascades are developed. One example of such cell-free processes was described for the enzymatic synthesis of ethanol and isopropanol starting from D-glucose [15]. Here, pyruvate is a central intermediate and produced through only five enzymatic steps, catalyzed by four different enzymes (Scheme 1).

The key enzyme in this cascade is a dihydroxyacid-dehydrogenase from *Sulfolobus solfataricus* (SsDHAD) which catalyzed not only the dehydration of D-gluconate to 2-keto-3-deoxygluconate, but also converted D-glycerate to pyruvate. Pyruvate was then further converted to produce ethanol and isobutanol. The core of this cascade has recently been adopted and modified to also produce other products like D-lactate and L-alanine [16,17]. Besides starting from D-glucose, the dehydratation of D-glycerate is also a critical step for cascades that convert glycerol into amino acids, for example [18]. Despite some optimization efforts, space-time-yields and conversion rates are currently too low to facilitate technical applicability due to the rather low activity of the SsDHAD for the conversion of D-glycerate. This dehydratase from the thermophilic archaeon *Sulfolobus solfataricus* incorporates an iron-sulfur cluster as cofactor and converts a broad range of dihydroxy acids [19,20]. Dehydratases generally represent central enzymes in the conversion of renewable resources into fuels and bio-based building blocks, as they are able to deoxygenate sugar-derived molecules. However, there are no enzymes described that specifically dehydrate D-glycerate with a high activity, as natural pathways utilize phosphorylated substrate variants. Nevertheless, SsDHAD showed at least some activity for this reaction, albeit at a very low level. Therefore, there is an urgent demand for the optimization of the D-glycerate dehydratase activity of this enzyme. Endeavors to improve the activity of a certain protein are, as discussed above, based on rational design approaches and random mutagenesis. Unfortunately, there is no crystal structure of SsDHAD available. Recently, however, crystal structures of an L-arabonate dehydratase from Rhizobium leguminosarum [21] and a D-xylonate dehydratase from Caulobacter crescentus [22] have been determined. Being members of the wider DHAD family, these sugar dehydratases comprise [2Fe–2S] clusters as proposed for SsDHAD [23,24]. The structure of a plant DHAD from *Arabidopsis thaliana* also revealed a [2Fe–2S] cluster at the active site [25]. Nevertheless, based on the unavailability of a high-resolution crystal structure of SsDHAD, we focused on optimizing the activity of SsDHAD for its substrate D-glycerate through a random mutagenesis approach. Thus, our first goal was the development of a high-throughput approach to facilitate the detection of improved D-glycerate dehydratases in mutant libraries.

## 2. Results

A rational design approach was, due to the unavailability of a crystal structure, severely limited. In combination with the fact that our primary goal was to enhance the conversion of the non-natural substrate D-glycerate, a random mutagenesis approach seemed more appealing to us. Consequently, we chose to apply error-prone PCR (ep-PCR). This random mutagenesis method relies on the reduction of the fidelity of a non-proofreading PCR polymerase by adding Mn^2+^ to the reaction buffer or using an unbalanced nucleotide mix. Under these conditions, the polymerase makes mistakes, and nucleotide changes in the DNA sequence are induced. The technique easily allows the generation of large mutant libraries, thus posing the need for a reliable and sturdy screening method that allows discriminating between the wildtype signal and potential hits, even at low starting activities.

Based on existing and, in part, commercially available colorimetric assays for the detection of pyruvate, we thus started to develop a screening method for the enzymatic dehydration of D-glycerate. As we envisioned screening a medium to large number of variants for their ability to produce pyruvate, the selected method should also allow a combination with an automated liquid handling system. Thus, we set out to design a screening method based on a traditional microplate assay, combined with handling of reaction components and crude cell extracts by the aid of a robotic system and excluding extensive and time- or cost-intensive enzyme purification steps.

### 2.1. Development of a Peroxidase-Based High Throughput Screening Method

**Figure 1:**
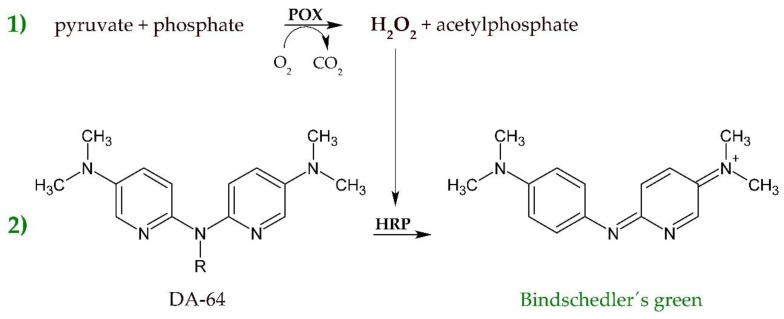
Coupled enzymatic and photometric assay for the detection of pyruvate. In a first step (1), H_2_O_2_ is released by pyruvate oxidase (POX) acting on pyruvate and phosphate. H_2_O_2_ is then consumed by horseradish peroxidase (HRP) to convert DA-64 to the green dye Bindschedler´s green (2), which can be quantified at 727 nm.

The starting activity of SsDHAD for the dehydration of D-glycerate was rather low [19]. To account for this situation, we first chose among available methods for the quantification of pyruvate. Pyruvate, the product of the enzymatic dehydration of D-glycerate, can be quantified by several methods, including coupled enzymatic reaction systems, chemical derivatization into colorant products, HPLC coupled with colorimetric or fluorimetric detection, as well as amperometric biosensors [26,27,28]. A very well-known example is the enzymatic reduction of pyruvate to D-lactate catalyzed by lactate dehydrogenase. Upon reduction of pyruvate, NADH is stoichiometrically converted to NAD^+^. This decrease in NADH concentration is proportional to the pyruvate concentration and measured spectrophotometrically. Using this assay at optimized conditions allows a limit of detection of about 0.3 µM pyruvate [28]. Methods with an even higher sensitivity rely on the coupling of two enzymes, where the first step is the release of hydrogen peroxide through the action of pyruvate oxidase and the second step is the conversion of a leuco dye by horseradish peroxidase to yield colorant products [28]. Many different dyes with similar sensitivity are described for the horseradish peroxidase reaction, with amplex red, 2,2-Azino-bis-3-ethylbenzothiazolin-6-sulfonic acid (ABTS), and DA-64, among others [26,27,28]. In principle, the formation of pyruvate from D-glycerate is easily followed by both of the abovementioned general methods, with the most convenient being the widely applied reduction of pyruvate to D-lactate by the action of lactate dehydrogenase and measuring the decrease of NADH absorption using a plate reader. We thus started the development of a screening protocol for D-glycerate dehydratase activity by expressing SsDHAD in 96 deep well plates. After harvesting and lysis, we applied the cell extracts for the conversion of D-glycerate and subsequently tried to detect generated pyruvate concentrations based on a standard LDH assay. Unfortunately, the low enzymatic starting activity of SsDHAD resulted in a very high variation of absorbance values, which we also attributed to inherently different cellular pyruvate and cofactor concentrations. Measuring the decrease of NADH concentrations is problematic, as any residual cellular activity that consumes NADH will directly lead to false positive hits. In order to get a selective and reliable detection signal for enzymatically produced pyruvate, we thus evaluated a coupled enzyme assay that converts pyruvate by simultaneously forming a colorimetric by-product, which is not naturally present in cell extracts. We chose the assay consisting of pyruvate oxidase, horseradish peroxidase, and DA-64 as leuco dye, which would subsequently develop a green-colored product by coupling the formation of pyruvate to the formation of Bindschedler’s green, which can be detected at 727 nm [27]. Tests with a standard solution of pyruvate showed a high sensitivity and a large dynamic range that would help to reduce the background signal as well as to enhance the detection quality. Figure 1 shows the reaction steps of this assay. It has the advantage of forming a well-detectable and stable dye and simultaneously offering high sensitivity and linearity at low concentrations of pyruvate.

In order to test the general assay strategy further and to modify it to an automated microplate-screening format, which would pose the opportunity of a sufficiently high throughput for screening a larger library of variants, we again expressed wildtype SsDHAD at a scale of 1 mL and harvested and lysed the cells. After centrifugation to remove cell debris, we applied different volumes of supernatant in enzymatic dehydration reactions and analyzed the linearity of the colorimetric assay. Unfortunately, the variation of the detection signal even among identical samples was extremely high and a comparison with the signal of purified enzyme preparations showed that there was almost no correlation between produced pyruvate and the signal of the assay for cell extract samples.

### 2.2. Effect of Media Components on the Peroxidase-Based D-Glycerate Dehydratase Assay

**Figure 2:**
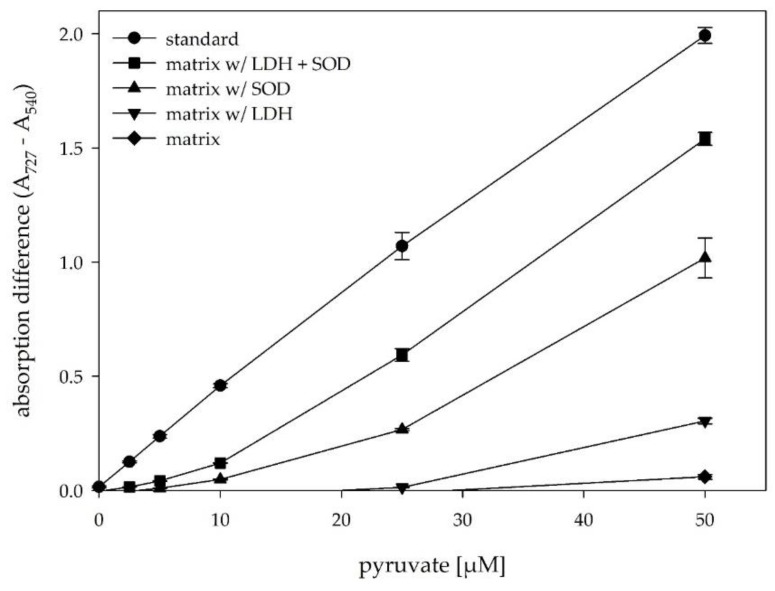
Effect of different enzymatic pre-treatment steps on the absorption difference in the presence of *E. coli* cell lysate. standard = pyruvate in water; matrix = *E. coli* cell lysate spiked with pyruvate, matrix w/ LDH = *E. coli* cell lysate, pre-treated with 12 U/mL LDH (30 min, 37 °C, 300 rpm); matrix w/ SOD = *E. coli* cell lysate, pre-treated with 35 U/mL SOD (30 min, RT, 300 rpm); matrix w/ LDH + SOD = *E. coli* cell lysate, pre-treated with LDH and SOD steps. A heat inactivation step was performed for all samples before adding the different pyruvate concentrations.

Various components in common media, nutrition components, precursors, and by-products might influence the peroxidase-based D-glycerate dehydratase assay, by e.g., inhibiting one of the enzymes or converting substrates to other products. Pyruvate is not only the product of the desired enzymatic activity, but also a naturally occurring metabolite and therefore present in variable concentrations within the cell lysates. This fact led to high signal variation even for samples not expressing SsDHAD (empty vector control). To reduce the background signal, we wanted to reduce the concentration of intrinsic pyruvate prior to setting up the enzymatic dehydration reaction. We decided to add lactate dehydrogenase to cell lysate. This enzyme would reduce pyruvate concentrations depending on NADH, which naturally occurs in the cell lysate. Subsequently measured pyruvate concentrations would then directly scale with the activity of SsDHAD for the conversion of D-glycerate. Surprisingly, we found that the signals for LDH pre-treated samples were significantly higher compared to the untreated samples and realized that our enzymatic detection assay was almost completely inhibited by the presence of *E. coli* BL21(DE3) cell lysate. For further evaluation of this phenomenon, we spiked cell lysate with different concentrations of pyruvate and analyzed the response of the enzymatic assay. Figure 2 shows that none of these samples gave an absorption signal above background at Bindschedler’s green wavelength. As expected from the previous experiment, the addition of LDH indeed increased the signal for samples with relatively high concentrations of pyruvate, although intended to decrease the metabolic pyruvate concentration by which it should decrease the absorption signal. To get some more insights into the mechanism of this unexpected behavior, we had a closer look on the reactivity of the dye Bindschedler’s green. This dye can be reduced back to the colorless Bindschedler’s green leucobase by chemical reduction agents. We supposed that NADH could also act as a reduction agent for Bindschedler’s green. As expected, standard samples of pyruvate spiked with NADH showed a reduced signal in the assay (see Supplementary Figure S1). Consequently, the increased signals for samples pre-treated with LDH were caused by a reduction of the NADH concentrations through the action of LDH. In order to get a more detailed picture of the assay inhibiting effect of cell lysate, we analyzed further samples that were spiked with different concentrations of pyruvate (Figure 2). Again, the pre-treatment of cell extract with LDH led to higher absorption values, nevertheless these were still too low by a factor of six compared to standard samples. Pyruvate concentrations below 25 µM did not give a signal at all. We assumed that the reducing effect of the cell lysate was still too high and tested the addition of superoxide dismutase (SOD) to the cell lysate instead of LDH.

SOD naturally converts superoxide radicals to hydrogen peroxide and was also described to inhibit the reduction of nitro blue tetrazolium through superoxide anions that occur during the oxidation of reduced metabolites with O_2_ [29]. We supposed that the addition of SOD would reduce the reductive potential of cell lysates by catalyzing the removal of superoxide radicals generated through the oxidation of NADH and other reducing substances. In fact, the addition of SOD allowed a much better detection of pyruvate in cell lysate and increased the signal by one order of magnitude. Even though the signal for pyruvate standard samples in water without cell lysate was still higher, the combination of LDH and SOD pre-treatment steps increased the absorption signal markedly. Application of the developed pre-treatment steps should thus enable the quantitative discrimination of pyruvate concentrations formed by SsDHAD reactions even in the presence of cell extract.

### 2.3. High-Throughput Screening for Improved D-Glycerate Dehydratase Activity

Encouraged by our results, we again analyzed samples expressing wildtype SsDHAD and performed the LDH and SOD pre-treatment steps. After the SOD step, we incubated at 70 °C for 30min to inactivate both LDH and SOD as well as other natural *E. coli* proteins. We then further applied such treated cell lysates to catalyze the reaction of D-glycerate to pyruvate in 96-well microtiter plates. After incubation for 90 min, we centrifuged the samples in the microplates to reduce the absorption background by removing denatured protein particles and transferred the supernatants to a fresh plate in order to perform the detection reaction forming Bindschedler´s green. After another incubation step for 45 min, the formation of the dye was accomplished and we quantified the absorption via photometric measurement at a wavelength of 727 nm against a background signal at 540 nm. On the basis of our optimization of the assay procedure, we could largely reduce the variation of absorbance signals and we further optimized all pipetting steps for the use of an automatic liquid handling platform, which could speed up the analysis as well as enhance the throughput.

**Figure 3:**
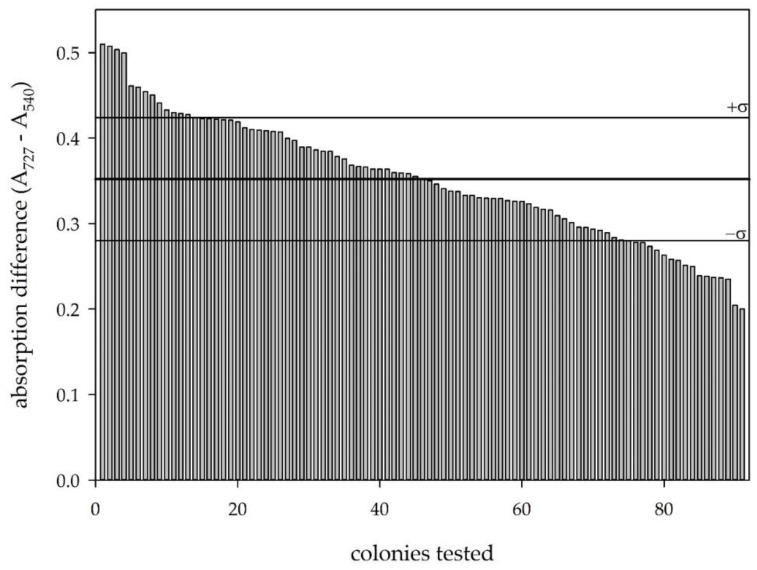
Wildtype landscape based on the developed screening protocol. The thick line shows the average of all values; the thin lines show the outer limits by the standard deviation.

To get a clearer picture of the applicability of the developed screening protocol for engineering D-glycerate dehydratases, we analyzed 96 separate clones expressing wildtype SsDHAD (Figure 3). Although we had performed some optimization on the screening protocol by pre-treatment of the cell lysate, the variation of our wildtype landscape with clones expressing wildtype SsDHAD was still high showing a mean absorption difference of 0.352 with a standard deviation of 0.072. The empty vector control showed values of 0.160 +/− 0.002, so that samples expressing wildtype SsDHAD showed a clear difference to the empty vector control.

**Figure 4:**
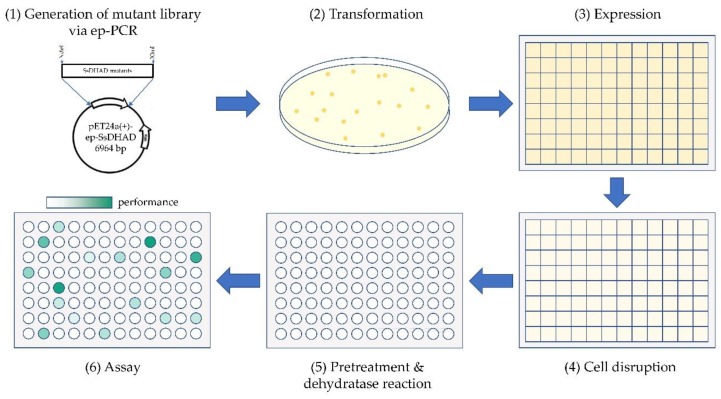
Schematic description of the screening method. (1) Generation of a mutant library by ep-PCR based on the SsDHAD gene and cloning into pET24a(+). (2) Transformation of *E. coli* BL21(DE3). (3) Single colonies were picked to inoculate a 96-deepwell plate as preculture. The preculture was diluted by a factor 50 for inoculation of a main culture. Expression was induced by IPTG after 3 h. (4) Cells were harvested and lysed by the action of lysozyme and ultrasonic. (5) Pre-treatment of cell extract by incubation steps with LDH and SOD followed by a heatstep. Samples were further applied for converting D-glycerate to pyruvate. (6) The produced pyruvate amounts were quantified through a coupled enzymatic and photometric assay and positive hits were selected with comparison to the pyruvate amounts of a wildtype landscape. Liquid handling steps were performed by automated liquid handling systems.

A widely accepted parameter for the qualitative evaluation of a screening method is the Z’-factor [30]. This factor is a dimensionless number that shows the discrimination of active clones from inactive ones by determining the signal difference between positive and negative controls in relation to the sum of their standard deviations. Screening protocols that show Z’-values between 0.5 and 1 provide reliable screening conditions, whereas assays with Z’ < 0.5 cannot distinguish between positive and negative clones with high precision. In our case, calculation of this Z´-factor gives only a very small value because of low absorption values combined with standard deviations that were still high despite some optimization. Nevertheless, the aim of our screening method was to select improved variants of the SsDHAD that show a higher activity compared to the wildtype enzyme. Because of this, the Z´-factor shows only limited practicability, as real positive clones are not available during method development. During the development of our screening, we could also identify conditions that led to Z’ values ranging between 0.6 and 0.8. However, to reach these values, we had to increase the signals of wildtype SsDHAD to very high values around 1.5. As the starting activity is no positive control regarding an enzyme engineering approach, we decided to accept a rather high relative variation and a bad separation of media controls and wildtype colonies, in order to keep a large selection window for positive variants. We therefore chose variable reaction parameters (e.g., volume of applied pre-treated cell lysate) to get rather low absorption difference for wildtype samples, in order to keep a large selection window for improved variants (Figure 4).

**Figure 5:**
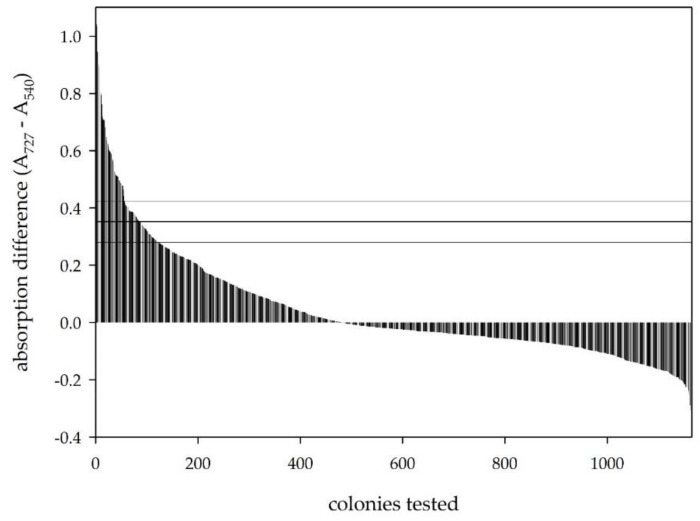
Screening results of almost 1200 clones of a SsDHAD mutant library. Positive clones were selected by the upper limit of the standard deviation of a wildtype landscape, shown by the horizontal lines.

After demonstrating the feasibility of the developed screening protocol, we aimed at its application in the high-throughput screening of mutant libraries of SsDHAD generated by random mutagenesis. For this, we created a mutagenic library of SsDHAD by ep-PCR. Sequencing of randomly selected mutants showed a frequency of 1 to 3 mutations per SsDHAD gene (i.e., per 1.67 kbp; this mutation rate was intended; the conditions were identified based on the preparation of small libraries with varying concentrations of Mn^2+^). In the first round of screening, we tested about 1200 variants for their activity on D-glycerate using the developed screening protocol with pre-treatment of the cell lysates (Figure 4). We selected hits based on an absorption difference higher than the upper standard deviation limit of wildtype clones that were co-processed with the mutant variants (Figure 5).

**Figure 6:**
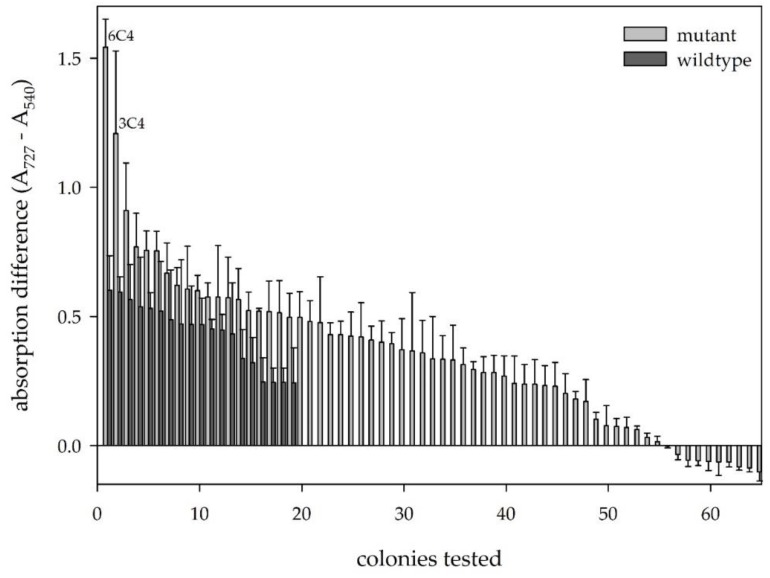
Re-screening of selected hits of the first round of screening. Dark = wildtype clones; light = rescreened mutants.

We thus identified 65 clones as first hits and applied those in a rescreening comprising four separate replicates of each hit. Most of the primarily selected variants showed absorption differences that were in the same range or even lower as the wildtype clones (Figure 6), but two variants showed a significantly higher signal and were chosen for further evaluation.

Sequencing of the two selected variants of the rescreening showed the same mutation. At position 1616, T is changed to C, which results in an amino acid change of leucine to proline at amino acid position 539. In addition to this mutation, one variant showed an additional mutation of the DNA sequence that is silent and did not lead to an altered amino acid sequence, but proves that the two selected variants differ based on their DNA sequence and represent individual clones instead of artefacts. Nevertheless, both selected variants produce SsDHAD with identical amino acid sequence and thus demonstrate the effectiveness of the screening protocol. In order to further analyze our screening protocol now with a real positive control at hand, we grew one of the selected variants (6C4) and wildtype SsDHAD in flasks, lysed the cells, and pre-treated the extracts identically to the screening protocol with LDH and SOD. For the D-glycerate reaction, we diluted the samples 1:100 and detected formed pyruvate by the D-glycerate dehydratase activity assay. As a result, variant 6C4 produced 40% more pyruvate compared to the wildtype and proves that our screening protocol is able to discriminate between positive and wildtype clones.

### 2.4. Analysis of Enzymatic Properties of the Selected D-Glycerate Dehydratase Variant

In order to characterize the selected variant and to compare its catalytic properties to the wildtype enzyme, we performed preparative expressions in *E. coli* BL21(DE3) and also included an activation step by adding 2-mercaptoethanol prior to a heat step at 70 °C. This activation was described to increase the specific activity by a factor of two [19]. We subsequently removed the 2-mercapto ethanol as well as small cellular compounds by a desalting step that concomitantly changed the buffer 5 mM HEPES. Wildtype and selected variant 6C4 showed comparable expression rates and were both purified to homogeneity. We then analyzed the catalytic properties of the enzyme preparations by incubation with variable concentrations of D-glycerate at 50 °C and measured the resulting pyruvate concentrations with HPLC. By following our protocol, wildtype showed an activity for the dehydration of D-glycerate of 48.4 +/− 2.0 mU/mg, whereas variant 6C4, showed a value of 70.9 +/− 1.8 mU/mg, which is 1.5 times higher compared to the wildtype (the enzyme unit (U) is defined as the amount of enzyme that catalyzes the conversion of one micromole of substrate per minute). We were interested whether this activation was due to a preferred conversion of the substrate D-glycerate or whether 6C4 generally shows higher dehydration rates. Thus, we also tested the conversion of D-gluconate to 2-keto-deoxy gluconate and found that 6C4 shows again higher activity with 1.6 U/mg compared to 1.3 U/mg for the wildtype enzyme. Wildtype SsDHAD is a thermostable enzyme and we wanted to test whether the activation of 6C4 had a detrimental effect on its stability. Although we already knew that it withstands a heat step at 70 °C, we performed a thermofluor assay to determine the stability against thermal denaturation [31]. For the wildtype, the determined melting temperature is 89.0 °C, while for the mutant 6C4, we measured a lower temperature of 82.0 °C. Variant 6C4 showed a slightly reduced thermal stability and we further characterized both enzymes regarding their thermal inactivation kinetics. We incubated both enzymes at a temperature of 50 °C and determined the thermal inactivation by taking samples of the enzymes at certain times points. By this, we could determine a half-life value for the wildtype of 3.4 h while the variant 6C4 showed a higher stability with a half-life of 5.1 h, despite its lower thermal melting point. In sum, the half-lives and stabilities of both enzyme variants are quite similar, but 6C4 shows an increase in activity for the screening substrate D-glycerate as well as for the substrate D-gluconate (Table 1).

## 3. Discussion

Enzymatic conversion of D-glycerate to pyruvate using dehydratases is a central reaction for the conversion of biomass-derived sugars into bulk and fine chemicals. In order to optimize the enzyme SsDHAD that naturally shows a promiscuous, but small activity for the dehydration of D-glycerate, through a random mutagenesis approach, we were aiming at the development of a high-throughput screening for D-glycerate dehydratase activity in cell extracts. A coupled enzymatic assay with pyruvate oxidase and horseradish peroxidase showed high sensitivity, but only with purified samples. Cellular levels of NADH reduced Bindschedler’s green back to its leuco-form and decreased the measured signals to background level. Pre-treatment steps with LDH and SOD enhanced the assay signal in presence of cell extract to a level that reliably supported the selection of positive hits. Incubation of cell extract with LDH not only reduced the cellular pyruvate content, but also decreased NADH levels and thus reduced the back reduction of Bindschedler’s green to its leucoform. SOD is generally applied to reduce the oxidative potential of superoxide radicals, but is also able to inhibit reductive reactions. Here, the addition of SOD markedly increased the assay signal and facilitated the proportionality between assay signal and pyruvate concentrations. We attribute this effect to a diminished reductive potential of pre-treated cell extracts through the conversion of superoxide to hydrogen peroxide, which we decompose during the heat precipitation step. In summary, the pre-treatment steps decrease the endogenous pyruvate concentration and reduce the reductive potential of the cell extracts. With this optimized assay for detecting pyruvate concentrations in the presence of cell extract at hand, we could successfully implement a microplate assay for the peroxidase-based analysis of D-glycerate dehydratase activity. We believe that our optimization approach is directly applicable to other peroxidase-type assays and that numerous enzymes could be optimized using this approach starting from a relatively low starting activity.

Despite some optimization on the analysis and screening of D-glycerate dehydratase activity, we were still not able to discriminate sharply between background and wildtype samples, which we attributed to the inherently high variation among wells. SsDHAD is inhibited by high substrate concentrations and has only a limited half-life [19]. Because of this, the enzymatic reaction cannot be optimized using higher substrate concentrations or prolonged reaction times. Consequently, a high variation of enzymatic activity is a natural feature of this class of enzymes and we were not able to decrease the signal variation by further optimizing the assay and screening protocols without a severe reduction of the selection window. Nevertheless, on the basis of our developed screening protocol, we could successfully show that beneficial selection is possible even with relatively low Z’-values, i.e., bad separation between positive and negative variants (or in our case wildtype and negative variants). Rather low enzymatic starting activities pose the need for significantly large selection windows. In our case, we optimized our assay for high reproducibility, but also for preserving a considerably large selection window. Because of this and a comprehensive rescreening, we were able to select beneficial variants out of a pool of promising variants.

Both selected variants showed identical sequences on the amino acid level and we characterized variant 6C4 in comparison to the wildtype enzyme. Purified 6C4 showed increased activity on D-glycerate as well as on D-gluconate at increased stability against thermal inactivation at 50 °C and only a small decrease of the melting temperature. These results, and the fact that the amino acid exchange is located distant from the active site, suggest that the activation is due to an increased flexibility. Despite the large increase in enzymatic activity, the reached values are still not appropriate to boost enzymatic cascades that rely on D-glycerate dehydration. Because of this, further library selection steps need to be performed and beneficial mutations combined in a combinatorial way to reach desired values. Nevertheless, the screening method developed within this work provides a valuable tool for this endeavor. Besides that, other peroxidase-based screening systems may also benefit from our results.

## 4. Materials and Methods

### 4.1. Chemicals and Enzymes

Restriction enzymes were obtained from New England Biolabs (Frankfurt am Main, Germany). Oligonucleotides were synthesized by Eurofins Genomics Germany GmbH. D-glycerate was purchased as D/L-glycerate from TCI Deutschland GmbH. Superoxide dismutase (SOD), pyruvate oxidase (POX), and horseradish peroxidase (HRP) were obtained from Sigma Aldrich (Saint Louis, MO, USA). Lactate dehydrogenase (LDH) was obtained from Carl Roth GmbH. The leuco dye N-(carboxymethylaminocarbonyl)-4,4′-bis(dimethylamino)-diphenylamine sodium salt (DA-64) was acquired from Wako (Neuss, Germany). All other chemicals were of analytical grade and purchased from Sigma-Aldrich (Taufkirchen, Germany), Carl Roth GmbH (Karlsruhe, Germany), Serva Electrophoresis GmbH (Heidelberg, Germany), Merck KGaA (Darmstadt, Germany), and Thermo Scientific GmbH (Darmstadt, Germany).

### 4.2. Cloning of the SsDHAD Gene and Generation of an SsDHAD Mutant Library

The DHAD sequence of *Sulfolobus solfataricus* (GenBank accession no.: NP_344419.1) was synthesized (Geneart, Regensburg, Germany) and cloned in pET24a(+) via NdeI and XhoI restriction sites. A mutant library was generated via ep-PCR using 1 mM Mg^2+^ and 0.25 mM Mn^2+^ combined with an unequal dNTP-mix (dATP 1.52 mM, dCTP 1.74 mM, dGTC 0.87 mM, dTTP 5.87 mM) [32], 1 ng/µl SsDHAD in pET24a(+) as template, and the oligonucleotides CATATGCCTGCAAAACTGAATAGC and CTCGAGTTATGCCGGACGGGTAACTG as primers. The mutant genes were again cloned in pET24a(+) via NdeI and XhoI restriction sites and a mutant library was created via transformation of *Escherichia coli* NEB turbo, followed by subsequent plasmid preparation via the GeneJET Plasmid Miniprep Kit (Thermo Scientific GmbH, Darmstadt, Germany).

### 4.3. Screening Method for Improved SsDHAD Variants

*Escherichia coli* BL 21(DE3) cells were transformed with the SsDHAD-pET24a(+) mutant library. Overnight cultures of transformed colonies were prepared in 96 deep well plates, which were grown for 48 h at 30 °C and then diluted by a factor of 50 upon inoculation of 1.2 mL main culture (LB medium containing 30 mg/L kanamycin). After incubation at 30 °C for 3 h, protein expression was induced by adding IPTG to a final concentration of 1 mM. After 16 h, cells were harvested by centrifugation (3000 *g*, 4 min, RT), resuspended in 400 µL potassium phosphate buffer (10 mM, pH 7.0, 0.1 mg/mL lysozyme), and incubated for 30 min at RT. Afterwards, the resuspended cells were disrupted by sonication (3 min, amplitude 60%, 420 W). Then, 7.5 U/mL LDH (30 min, 37 °C, 300 rpm) and 20 U/mL SOD (30 min, RT, 300 rpm) were added separately and the plates incubated for the indicated time period. Subsequently, a heat inactivation step (70 °C, 30 min, 300 rpm) was performed. Then, 10 µL of substrate D/L-glycerate (100 mM, pH 7.0) were added and the reaction mix incubated with shaking for 90 min at 50 °C. The supernatants after centrifugation (3000 *g*, 30 min, RT) were then analyzed by the D-glycerate dehydratase activity assay.

To determine the influence of pretreatment steps with LDH/SOD, samples were treated similar to the described screening. Heterologous protein expression of SsDHAD was again performed in *Escherichia coli* BL 21(DE3). Transformed strains were grown as 5 mL cultures (LB medium containing 100 mg/L kanamycin), which were inoculated by overnight cultures (dilution factor 1:50). After growing for 3 h (30 °C, 160 rpm), protein expression was induced by adding IPTG to a final concentration of 1 mM. Cells were harvested by centrifugation (3000 *g*, 4 min, RT) after additional 16 h and resuspended in 1.6 mL potassium phosphate buffer (10 mM, pH 7.0, 0.1 mg/mL lysozyme) and incubated for 30 min at RT. The cells were disrupted by sonication (3 min, amplitude 60% 420 W). As pretreatment, 12 U/mL LDH (30 min, 37 °C, 300 rpm) and/or 35 U/mL SOD (30 min, RT, 300 rpm) were added and the reaction mixtures incubated for the indicated period of time before proceeding with the next step. Subsequently, a heat inactivation step (70 °C, 30 min, 300 rpm) was performed. Then, 135 µL of H_2_O were added instead of substrate and the resulting mixtures were incubated for 90 min (50 °C, 120 rpm). After centrifugation (3000 *g*, 30 min, RT) 90 µL of the supernatants were spiked with different concentrations of pyruvate. The D-glycerate dehydratase activity assay was then performed as described below.

Automated liquid handling was performed using a Tecan Freedom EVO 200 platform with the Tecan Evoware Plus software and a Brand Liquid handling station flow with the software version 2.20.301. Detailed liquid and plate handling software scripts are available upon request.

### 4.4. D-Glycerate Dehydratase Activity Assay

Pyruvate concentrations were determined through a coupled enzymatic assay that stoichiometrically formed a colorant product. As the first step, hydrogen peroxide is produced as an intermediate by the action of pyruvate oxidase. In the second step, the leuco dye DA-64 is converted to Bindschedler’s green through action of horseradish peroxidase. To quantify pyruvate in a large number of samples, a master mix was prepared, containing 50 mM potassium phosphate buffer pH 5.6, 100 µM MgCl_2_, 50 µM DA-64, 50 µM thiamin pyrophosphate (TTP), 0.05 U pyruvate oxidase (POX), and 0.25 U horse radish peroxidase (HRP). Then, 100 µL of mastermix were mixed with 100 µL of sample and incubated for 40 min at 37 °C with shaking at a speed of 500 rpm. Bindschedler’s green was then detected at a wavelength of 727 nm and measurements corrected by background absorption at 540 nm.

### 4.5. Expression and Purification of SsDHAD Variants

Heterologous expression of SsDHAD and the selected variant 6C4 were performed in *Escherichia coli* BL 21(DE3) in shaking flasks with autoinduction medium ZYP-5052 supplemented with 30 mg/L kanamycin and 1 mg/L FeSO_4_ as described previously [19,33]. After inoculation of the autoinduction media with an overnight preculture (1:50), cells were again grown overnight (90 rpm, 22 °C), harvested by centrifugation (4000 *g*, 15 min, RT), and stored at −20 °C.

For purification of SsDHAD variants, pelleted cells were resuspended in HEPES-Buffer (100 mM, pH 8.0) at a concentration of 20% (*w*/*v*) and disrupted by sonication. The resulting crude extracts were activated by addition of 98 mM 2-mercaptoethanol and an incubation step at 70 °C for 30 min. After centrifugation (12,000 *g*, 30 min, RT) the supernatants were collected and the protein solutions desalted again 5 mM HEPES pH 7.5 via an Äkta pure chromatography system equipped with a HiPrep 26/10 desalting column (GE Healthcare, Freiburg, Germany).

### 4.6. Determination of Protein Concentrations and Purity

Protein concentrations were measured by a Bradford protein assay using the Roti-Quant reagent (Carl Roth GmbH, Karlsruhe, Germany) according to the manufacturer’s recommendations, using bovine serum albumin as standard. The purity of enzyme preparations was controlled using SDS-PAGE.

### 4.7. HPLC Analytics

HPLC measurements were performed on an Ultimate-3000 HPLC system (Dionex), equipped with an autosampler and a diode-array detector. Chromatographic separation of D-gluconate, KDG, pyruvatem and D-glycerate was achieved an a Metrosep A Supp10-250/40 column (250 mm, particle size 4.6 µm, Metrohm, Filderstadt, Germany) at 65 °C by isocratic elution with 12 mM ammonium bicarbonate (pH 10), followed by a washing step with 30 mM sodium carbonate (pH 10.4) [19]. Mobile phase flow was adjusted to 0.2 mL/min. Sample volume was 10 µL in each case. System calibration was performed using external standards. Samples were prepared by filtration (10 kDa MWCO, modified PES, VWR, Darmstadt, Germany) and diluted to a maximal sample concentration of 1 mM.

### 4.8. Enzymatic Activity for Dehydration of D-Glycerate or D-Gluconate

Enzymatic activity was analyzed in a discontinuous assay by incubating 600 µL of purified enzyme solution with 40 mM D/L-glycerate (titrated to pH 7.0) or 20 mM D-gluconate at 50 °C in a total volume of 1 mL. The reactions were stopped after 60 min by freezing in liquid nitrogen. Analysis of collected samples was performed after thawing as described above. For determination of kinetic parameters, the Michaelis–Menten equation was applied using Sigma Plot (version 13.0).

### 4.9. Thermal Stability

Thermal stability of SsDHAD variants was analyzed using a thermofluor assay [31]. Then, 2 µL of a 1:80 dilution of SYPRO™ Orange (Sigma Aldrich) in HEPES (5 mM, pH 7.8) and 2 µL of enzyme solution were mixed with buffer (5 mM HEPES pH 7.8) to reach a total volume of 20 µL. A temperature gradient was applied and fluorescence measurements (excitation at 485/20 nm, detection at 530/30 nm) conducted.

For thermal inactivation kinetics, aliquots of purified enzyme solution were incubated at 50 °C and the enzyme activities at certain time points were determined as described above.

## Figures and Tables

**Table 1:** Characterization of wildtype SsDHAD and selected variant 6C4.

Variant	Spec. Activity (D-Glycerate) [mU/mg]	Spec. Activity (D-Gluconate) [U/mg]	T_1/2_ [h]	Melting Temperature [°C]
Wildtype	48.4 +/− 2.0	1.29 +/− 0.03	3.4	89.0
6C4	70.9 +/− 1.8	1.62 +/− 0.07	5.1	82.0

+: Indicates the standard deviation of the determined values; −: Indicates the standard deviation of the determined values.

## Supplementary Materials

Supplementary materials can be found at https://www.mdpi.com/1422-0067/21/1/335/s1.

## Abbreviations

ABTS	2,2-Azino-bis-3-ethylbenzothiazolin-6-sulfonic acid
DHAD	Dihydroxyacid dehydratase
*E. coli*	*Escherichia coli*
ep-PCR	Error-prone PCR
LDH	Lactate dehydrogenase
NAD^+^, NADH	Nicotinamide adenine dinucleotide
SOD	Superoxide dismutase
SsDHAD	Dihydroxyacid dehydratase from *Sulfolobus solfataricus*
TTP	Thiamine pyrophosphate
U	Enzyme unit
