# Supplementary material for: Development of an Improved Peroxidase-Based High-Throughput Screening for the Optimization of D-Glycerate Dehydratase Activity
Source: Int J Mol Sci. 2020 Jan 3;21(1):335. doi: 10.3390/ijms21010335 (PMC6982167; doi:10.3390/ijms21010335)
Supplement: Supplementary file 1 [file ijms-21-00335-s001.pdf]

International Journal of
Molecular Sciences

Supporting information

High-Throughput Screening for the Optimization of D-Glycerate Dehydratase Activity

Benjamin Begander [1], Anna Huber [1], Manuel Döring [1], Josef Sperl [1] and Volker Sieber [1,2,3,*]

[1] Chair of Chemistry of Biogenic Resources, Campus Straubing for Biotechnology and Sustainability, Technical University of Munich, Schulgasse 16, D-94315 Straubing, Germany; benjamin.begander@tum.de (B.B.); anna97.huber@tum.de (A.H.); manuel.doering@tum.de (M.D.); josef.sperl@tum.de (J.S.)
[2] Catalysis Research Center, Technical University of Munich, 85748 Garching, Germany
[3] School of Chemistry and Molecular Biosciences, The University of Queensland, St. Lucia, QLD 4072, Australia
* Correspondence: sieber@tum.de; Tel.: +49-9421-187-300

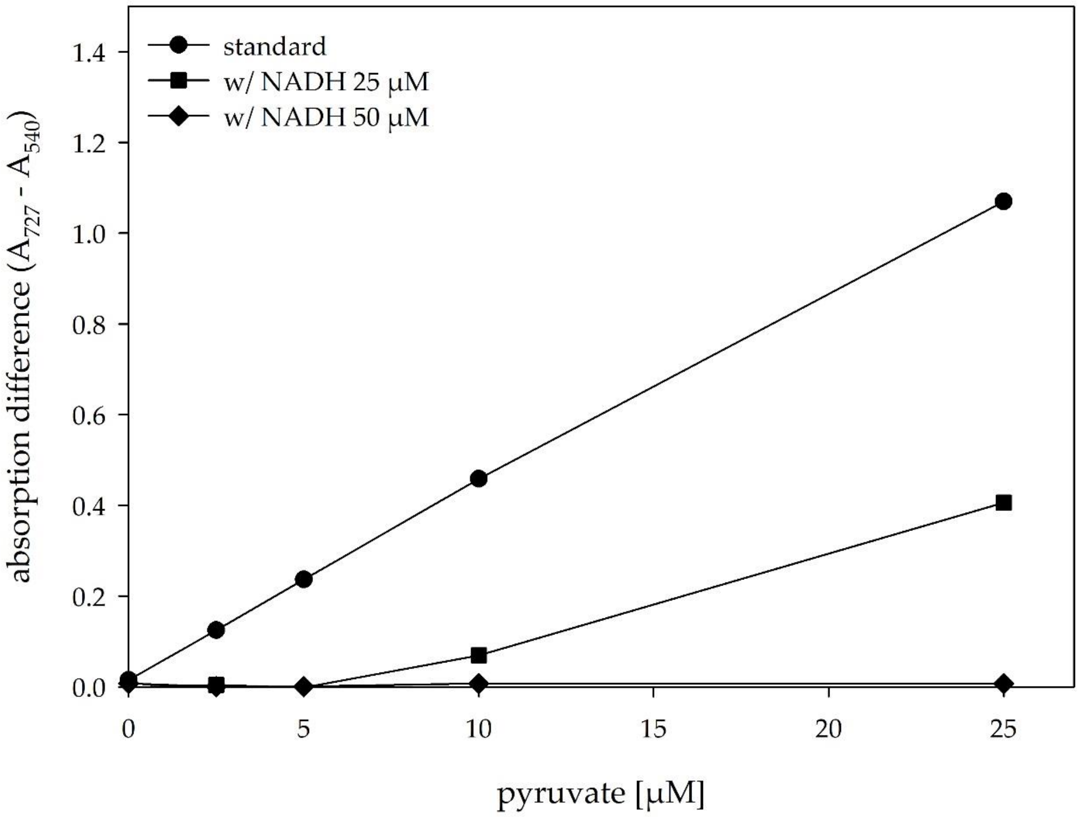

Figure S1. Effect of spiking with NADH on the absorption difference of pyruvate standard samples. standard = pyruvate in water; w/ NADH = pyruvate in water, spiked with the indicated concentration of NADH.

© 2019 by the authors. Submitted for possible open access publication under the terms and conditions of the Creative Commons Attribution (CC BY) license (http://creativecommons.org/licenses/by/4.0/).
